# Alterations in complement and coagulation pathways of human placentae subjected to in vitro fertilization and embryo transfer in the first trimester

**DOI:** 10.1097/MD.0000000000017031

**Published:** 2019-11-01

**Authors:** Liang Zhao, Lifang Sun, Xiuli Zheng, Jingfang Liu, Rong Zheng, Rui Yang, Ying Wang

**Affiliations:** a Department of Obstetrics and Gynecology, Beijing Jishuitan Hospital; b Department of Obstetrics and Gynecology, Peking University Third Hospital, Beijing, China.

**Keywords:** complement and coagulation pathways, first trimester, in vitro fertilization and embryo transfer (IVF-ET), placenta, pregnancy complication

## Abstract

The mechanisms underlying the potential risks of in vitro fertilization and embryo transfer (IVF-ET) have not been fully elucidated. The aim of this study was to explore changes in the complement and coagulation pathways in placentae subjected to IVF-ET in the first trimester compared to placentae from normal pregnancies. Four placenta samples in the first trimester were obtained from patients undergoing IVF-ET owing to oviductal factors only. An additional 4 control placentae were obtained from volunteers with normal pregnancies. A GeneChip Affymetrix HG-U133 Plus 2.0 Array was utilized to analyze the changes in gene expression between the normal and IVF-ET placentae. Differentially expressed genes (DEGs) were analyzed using the Database for Annotation and Visualization and Integrated Discovery bioinformatics resource, and gene ontology enrichment analysis and Kyoto Encyclopedia of Genes and Genomes (KEGG) pathway analysis were conducted. Using real-time PCR, we confirmed the obtained microarray data in 10 dysregulated genes. Five of the gene products were further analyzed by immunohistochemistry (IHC) to determine their protein expression and localization. A total of fifty DEGs were identified in the complement and coagulation pathways in the IVF-ET treated placentae: 38 upregulated and 12 down-regulated. KEGG pathway analysis indicated that IVF-ET manipulation substantially over-activated the coagulation and complement pathways, while urokinase plasminogen activator- and urokinase plasminogen activator receptor-mediated trophoblastic invasion and tissue remodeling were inhibited. Furthermore, the 5 proteins analyzed by IHC were found to be localized specifically to the placenta. This is the first study to compare DEGs relating to the placental complement and coagulation pathways from patients undergoing IVF-ET treatment compared to those undergoing normal pregnancy. These findings identified valuable biomarkers and potential novel therapeutic targets to combat the unfavorable effects of IVF-ET.

## Abstract 2

Supplemental Digital Content is available in the text

## 1 Introduction

Therapeutic strategies against infertility caused by various etiological factors have improved greatly in recent years, particularly in vitro fertilization and embryo transfer (IVF-ET).^[1]^ However, even after adjusting for several confounding factors, the risk of adverse outcomes during the perinatal period, including miscarriage, premature birth, low birth weight, intra-uterine growth retardation, and gestational hypertension, have been reported to be higher in IVF-ET cohorts than in subjects with spontaneous pregnancies.^[2,3]^ In recent years, the early stages of mammalian embryonic development have been found to be very sensitive to their microenvironment, with long-term effects on fetal, postnatal, and adult health.^[4,5]^ The concept of the developmental origins of health and disease, based on accumulating evidence that prenatal exposure to modified environmental conditions affects postnatal growth, metabolism, and disease susceptibility in adulthood, has been extended to the preimplantation stages of development.^[6]^

Recently, increasing research has shown that placental tissues are more sensitive than embryonic tissues to the preimplantation epigenetic dysregulation of imprinted genes.^[7,8]^ This can lead to abnormal placental development and function with possible consequences for the developing fetus. Based on this observation, subsequent studies have proposed 2 possible scenarios to explain why these defects appeared to be restricted to the trophectoderm lineage.^[9,10]^ On one hand, trophectoderm cells, which are in contact with the culture medium, are more strongly influenced by in vitro culture, which is responsible for a loss of imprinting in mid-gestation placenta.^[11]^ On the other hand, they are also the first lineage to differentiate in the embryo as trophectoderm stem cells, from which the different cell lines in the future placenta will originate.^[12]^ In addition to the fact that the composition of culture media differs from that of the in vivo natural environment, and despite careful manipulation, in vitro cultured trophectoderm cells are vulnerable to several environmental stressors, such as oxygen tension, pH and temperature variations during manipulation, light exposure, and shear stress linked to repeated pipetting, which may affect placental development and function.^[13]^

Increasing evidence supports the hypothesis that some adverse pregnancy outcomes observed after IVF-ET are due to suboptimal placenta function caused by abnormal trophoblastic invasion.^[14]^ Notably, trophectoderm cells from blastocysts cultured in vitro showed major changes in gene expression, including the activation of stress-related pathways and the down-regulation of genes involved in placentation.^[15]^ Furthermore, in human placentas after IVF-ET, genome-wide mRNA expression analysis identified the overexpression of genes involved in metabolism, immune response, transmembrane signaling, and cell cycle control.^[16]^ Similarly, transcriptome data in mouse placental tissues showed that some IVF techniques may trigger the induction of genes involved in cellular proliferation and cell cycle progression.^[17]^

The complement and coagulation systems are 2 evolutionarily conserved and closelt related proteolytic cascades in plasma that play important roles in host defense and hemostasis, respectively.^[18]^ Complement and coagulation proteins circulate in the blood as inactive precursors, but are activated upon contact with target structures. The resulting proteolytic cascade generates multiple protein cleavage products that trigger numerous events leading to the onset of inflammation and hemostasis.^[19]^ The parallel expression of activation products for the complement and coagulation systems has long been observed in both clinical and experimental settings. The coexistence and interplay of hemostatic and inflammatory mediators in the same microenvironment typically ensures a successful balance in the hemostatic system, leading to a state of hypercoagulability during normal pregnancy.^[20]^ However, the dysregulation of this cascade or the presence of inhibitors in one or both systems can lead to obstetric complications with critical thrombotic and/or inflammatory complications.^[21,22]^

In the present study, we hypothesized that the expression of genes relating to the complement and coagulation pathways is altered in placental tissue during the first trimester after IVF-ET compared to that in placental tissue from spontaneous pregnancies. Therefore, we performed a microarray analysis to investigate and discover the potential effects of IVF-ET treatment on placental gene expression relating to the complement and coagulation pathways during early stages. The aim of this study was to explore the possible causal relationship between IVF-ET and the increased frequency of adverse pregnancy outcomes. Furthermore, an improved understanding of placental mechanisms triggered by IVF-ET may be of value in future to improve the safety of IVF-ET protocols.

## 2 Methods

### 2.1 Study subjects

Between January 2017 and October 2018, twin to singleton fetal reduction was performed in 4 cases at a mean of 49 ± 6 days into pregnancy after IVF-ET treatment (age range: 23–35 years; mean age: 30.7 years). All patients were undergoing IVF-ET due to oviductal obstructions. The quality of each male sperm was confirmed to be normal. The clinical application of assisted reproductive technology (ART) was licensed by the Ministry of Health of the People's Republic of China. The control group comprised 4 cases of unwanted twin pregnancies in the same period (age range: 24–30 years; mean age: 28.9 years). Clinical data were collected by the Department of Obstetrics and Gynecology at Beijing Jishuitan Hospital and organized in a database. The sample size of 4 for the microarrays, which is the minimal number required for statistical validity, was determined based on the availability of samples meeting the study's diagnostic criteria. Each of the 4 placental specimens from the IVF-ET group was matched to a control sample based on maternal age, and gestational age.

### 2.2 Sample collection and ethics approval

All fetal reductions were performed by the same senior physician through fetal bud aspiration under B ultrasound guidance. Four cases were selected for villi suction at the same time. Tissues were collected 30 to 45 days after embryo transfer, which is equivalent to 45 to 50 days of pregnancy. Patients in the control group were diagnosed with early intrauterine pregnancy after a bimanual examination, urine pregnancy test, and B ultrasound. All the patients had regular menstruation cycles and had not taken any steroid hormone drugs in the previous 3 months. Villi were obtained during the conventional artificial abortion operation. The villi samples were immediately separated from the specimens within 1 hour under an inverted microscope. All samples were then suspended in ice-cold PBS and subsequently stored in liquid nitrogen until total RNA extraction. A section of the remaining specimen was immersed in 4% formalin for 24 hours and then removed and rinsed under running water for 30 minutes. The villi were then subjected to routine dehydration, wax dipping, embedding, and slicing. One slice was obtained via 2-step immunohistochemical PV-9000. The study design was approved by the Ethics Committee of Beijing Jishuitan Hospital, China (Permission no 201703-11). Written informed consent to participate in this study was obtained from each individual prior to recruitment. All study participants were recruited at the Beijing Jishuitan Hospital Beijing 2017 to 2018, and were of Asian ancestry and living in Beijing.

### 2.3 RNA extraction

Tissue homogenization and RNA extraction, as well as microarray analysis (described below), were performed by CapitalBio Corporation (Bejing, China). Tissue homogenization total RNA extraction were performed using the Macherey–Nagel NucleoSpin RNA II kit (Macherey–Nagel, Duren, Germany) according to the manufacturer's instructions. The RNA, extracted with ribosomal 28S and 18S RNA with a ratio of intensities of 1.5 to 1.8:1, was used for both the microarray assay and quantitative real-time PCR (qRT-PCR).

### 2.4 Microarray analysis

To compare the differentially expressed genes (DEGs) between the 2 groups, an aliquot (2 μg) of total placental RNA was used to synthesize cDNA, which was subsequently transcribed to biotin-tagged cRNA using the MessageAmp II aRNA Amplification Kit (Ambion Inc., Carlsbad, CA). The cRNA was then fragmented to produce 35 to 200-nt strands in accordance with the manufacturer's protocols (Affymetrix, Santa Clara, CA). Microarray analysis was performed using the Affymetrix Human Genome U 133 plus 2.0 GeneChip (about 54,675 probes covering more than 32,228 transcripts and variants, representing more than 20,000 genes mapped through UniGene or via RefSeq annotations) following the manufacturer's instructions. After purification and washing, samples were incubated at 94°C for 35 minutes to fragment the RNA, followed by incubation and hybridization of the labelled amplified RNA at 45°C for 16 hours.

The arrays were washed and stained with streptavidin-phycoerythrin in an Affymetrix GeneChipFluidics Station 450, and subsequently scanned on an Affymetrix GeneChip Scanner 3000 to analyze the hybridization data. The scanned images obtained were first assessed by visual inspection and then analyzed by Affymetrix GeneChip Operating Software (GCOS, v 1.4). To normalize the different arrays, dChip software was used with global scaling. For the comparative analysis, a 2-class, unpaired method developed in Significance Analysis of Microarrays software (SAM, v 3.02; Stanford University, Stanford, CA) was used to compare significantly DEGs in the IVF-ET and natural pregnancy groups.

### 2.5 Gene expression data analysis

The microarray data of analyzed placental samples are MIAME compliant, and the raw datasets were deposited in the Gene Expression Omnibus (GEO) data repository (n = 8, accession no. GSE 122214). Gene expression between IVF-ET and natural pregnancy placentas was compared and defined statistically by analysis of variance with a false discovery rate of 1%. Genes showing a fold change of > 2 and *P* < .01 were considered to be differentially expressed. Fisher exact test (*P* < .05) was used for canonical pathway analysis. To assess the function of identified DEGs, the functional analysis and clustering tool from the Database for Annotation, Visualization and Integrated Discovery (DAVID) resource was used, as it provides a comprehensive set of functional annotation tools for investigators to understand the biological significance of a large list of genes (http://david.ncifcrf.gov). Bioinformatics tools, including DAVID v 6.7, were used to perform gene ontology (GO) functional enrichment analysis and annotation of the identified DEGs. DAVID was used to search a block of functionally related genes according to different criteria, such as GO terms for biological process, cellular component, and molecular function. The Kyoto Encyclopedia of Genes and Genomes (KEGG) Orthology-Based Annotation System (KOBAS 2.0) (http://kobas.cbi.pku.cn) was employed to identify enriched KEGG pathways based on an adjusted *P* value. Search tool for the retrieval of interacting genes (STRING) software was used to draw the genetic interaction network (https://string-db.org/).

### 2.6 Microarray validation by real-time PCR

To validate the microarray results, 500 ng of the same RNA samples were reverse-transcribed using the PrimeScript RT reagent Kit (Perfect Real Time) (TaKaRa Biotechnology [Dalian] Co., Ltd., Dalian, China). Amplification reactions were conducted using SYBR Premix Ex Taq (Perfect Real Time) (TaKaRa Biotechnology [Dalian] Co., Ltd.) with an ABI PRISM 7300 system. Transcripts encoding fibrinogen beta chain (*FGB*), fibrinogen alpha chain (*FGA*), fibrinogen gamma chain (*FGG*), serpin peptidase inhibitor, clade C (antithrombin), member 1 (*SERPINC1*), protein C (inactivator of coagulation factors Va and VIIIa) (*PROC*), plasminogen activator urokinase (*PLAU*), plasminogen activator urokinase receptor (*PLAUR*), serpin peptidase inhibitor, clade E (nexin, plasminogen activator inhibitor type 1), member 1 (*SERPINE1*), *CD59*, and complement factor D (adipsin) (*CFD*) were used for microarray validation. The primers used for qRT-PCR are listed in Supplementary Table S1. qRT-PCR was conducted on the above 10 genes that were found to be differentially expressed based on microarray analysis; furthermore, their functions were considered to be closely related to critical placental functions based on biological function analysis. The same RNA samples were used as those extracted from placental samples. The analysis of differences in gene expression between the study groups was performed using the Mann–Whitney *U* test. A value of *P* < .05 were considered statistically significant.

### 2.7 Immunohistochemistry

To improve our understanding of the functions of these confirmed DEGs in controlling the complement and coagulation pathways in placenta in the IVF-ET and control groups, immunohistochemistry (IHC) was performed to analyze the activity of the related proteins using 4-μm sliced placental paraffin sections. The placental tissue samples used for IHC staining originated from the same tissue block as those collected for RNA extraction. The following primary antibodies were used for IHC: FGB (ab232793), FGG (ab217783), SERPINC1 (ab126598), PLAU (ab133563), and PLAUR (ab82220, Abcam, Cambridge, UK). Formalin-fixed, paraffin-embedded placental tissues from the control group were deparaffinized, re-hydrated, and sectioned into 4-μm slices. All IHC staining has conducted according to the manufacturer's instructions. Imaging was performed with the Olympus BX60 microscope using Olympus DP71 digital camera and CellA imaging software (Olympus Optical, Tokyo, Japan).

## 3 Results

### 3.1 Patient characteristics

There were no statistical differences between the 2 groups of women in terms of age, gestational age, or body mass index (Table 1).

**Table 1:**
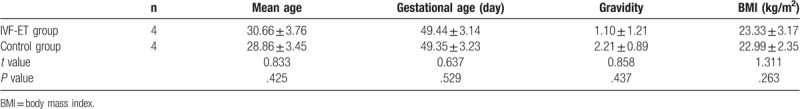
Characteristics of the patients in the 2 groups.

### 3.2 Cluster analysis of microarray data

**Figure 1:**
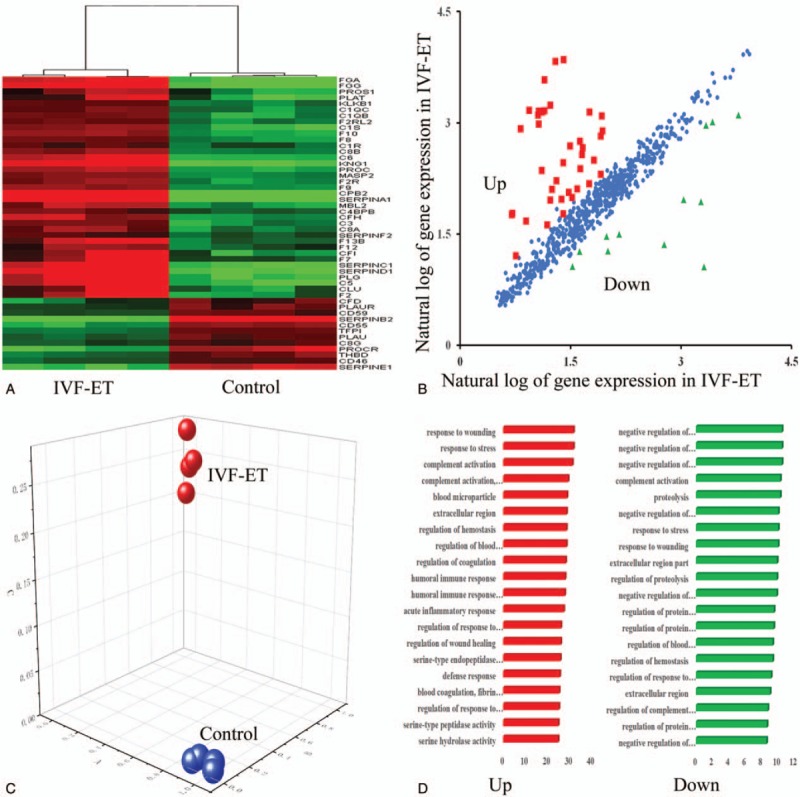
Clustering and principal component analysis of differentially expressed genes related to the complement and coagulation pathways in placentae after in vitro fertilization and embryo transfer (IVF-ET) in the first trimester compared to placentae from natural pregnancies. (A) Hierarchical clustering of 50 differentially expressed genes between IVF-ET and natural pregnancy placenta samples. (B) Scatter plot showing the changes in expression of all differentially expressed genes relating to the complement and coagulation pathways in the placenta after IVF-ET. (C) Principal component analysis of the 50 differentially expressed genes. (D) Functional enrichment analysis of the differentially expressed genes during first trimester according to biological processes.

A total of fifty DEGs related to the complement and coagulation signaling pathways were identified. Among them, 38 transcripts were up-regulated, and 12 transcripts were down-regulated in the placenta during the first trimester between the IVF-ET and natural pregnancy samples. Interestingly, the majority of DEGs were up-regulated (76%), while only 24% of the genes were down-regulated in the early placenta after IVF-ET treatment (Fig. 1A and B, Table 2). Hierarchical clustering was applied to the microarray data and a very clear separation was observed in the gene expression profiles between IVF-ET and natural pregnancy samples in the same period; the 2 groups were distinctly clustered into 2 different groups (Fig. 1A). In addition, principal component analysis demonstrated a similar pattern between the IVF-ET and natural pregnancy placental gene expression (Fig. 1C).

**Table 2:**
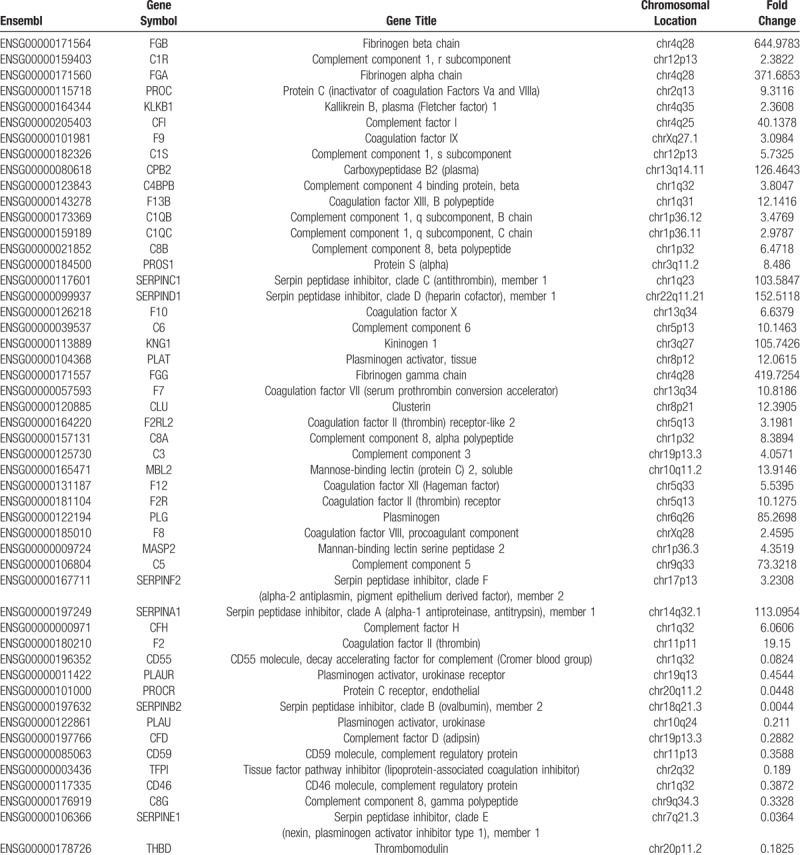
Genes differentially expressed in placenta derived from IVF-ET treatment.

### 3.3 GO pathway and network analysis

**Figure 2:**
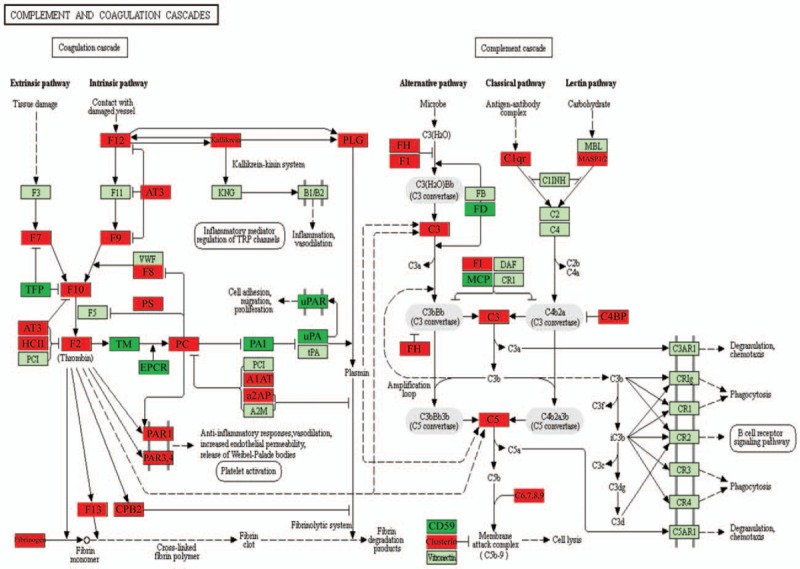
Schematic representation of genes in the placental complement and coagulation pathways affected by in vitro fertilization-embryo transfer (IVF-ET). All the differentially expressed genes analyzed here were found to affect the placental complement and coagulation pathways in IVF-ET samples during the first trimester. Red represents up-regulation; dark green represents down-regulation; light green represents no significant change.

The functional enrichment analysis of DEGs related to the complement and coagulation signaling pathways revealed that the observed genes also participated in more than 200 statistically over-represented GO categories (Fig. 1D). All DEGs and their functions in the complement or coagulation signaling pathways are shown in Figure 2. For the complement pathway, the up-regulation of genes relating to the classical, lectin, and alternative pathways was observed. Crucial extrinsic pathway, intrinsic pathway, and fibrinolytic genes within the coagulation system were widely up-regulated, whereas important genes relating to cell adhesion, invasion, migration, and proliferation were down-regulated (Fig. 2). In addition to participating in the complement and coagulation signaling pathways, these 50 DEGs also participated in multiple other broad signaling pathways, including *Staphylococcus aureus* infection (n = 13, false discovery rate [FDR]: *P* = 2.11 × 10^−23^), systemic lupus erythematosus (n = 10, FDR: *P* = 2.02 × 10^−13^), prion diseases (n = 7, FDR: *P* = 2.03 × 10^−12^), pertussis (n = 7, FDR *P* = 2.69 × 10^−10^), Chagas disease (American trypanosomiasis) (n = 4, FDR: *P* = 6.83 × 10^−5^), platelet activation (n = 4, FDR: *P* = 1.27 × 10^−4^), neuroactive ligand-receptor interaction (n = 4, FDR: *P* = 2.37 × 10^−4^), phagosome (n = 3, FDR: *P* = .36%), hematopoietic cell lineage (n = 2, FDR: *P* = 1.32%), and AGE-RAGE signaling pathway in diabetic complications (n = 2, FDR: *P* = 1.85%) (Supplementary Table S2).

**Figure 3:**
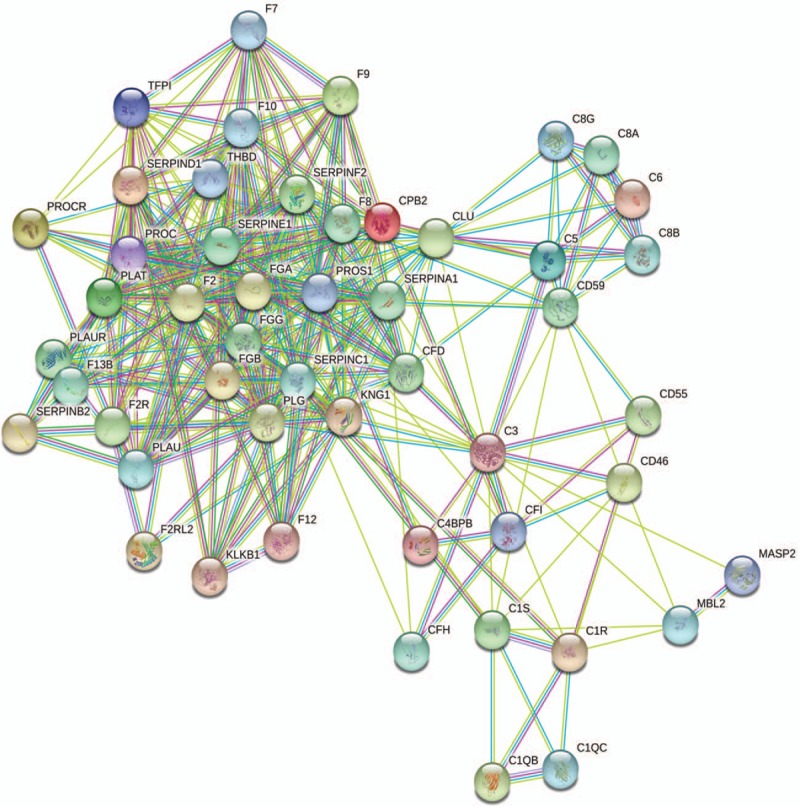
Gene interaction networks in placental complement and coagulation pathways affected by in vitro fertilization-embryo transfer (IVF-ET) in the first trimester. All 50 differentially expressed genes were used as input for STRING analysis and a network was built. Differentially expressed genes based on high confidence are shown.

The 50 DEGs were also mapped using STRING online software (Fig. 3). Their transcripts were widely distributed in the nucleus, cytoplasm, and cell membrane of placental cells. Bioinformatics analyses of the data suggest that various molecular and cellular functions were affected, and show their link to pregnancy complications.

### 3.4 Validation by real-time PCR

**Figure 4:**
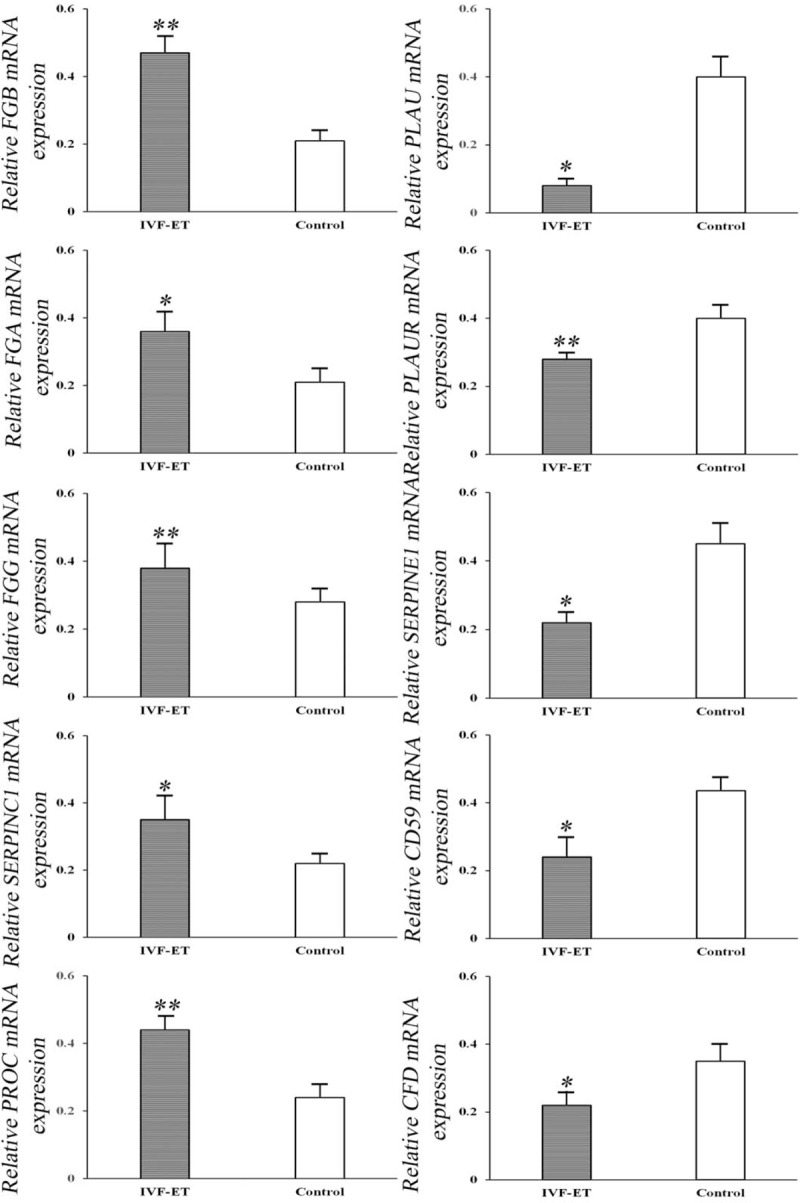
Validation of differentially expressed genes by real-time (RT)-PCR. Ten genes were subjected to RT-PCR to confirm their differential expression in placentae derived from IVF-ET samples compared to those from control samples in the first trimester. ∗ *P* < .05, ∗∗ *P* < .01.

To validate the microarray results, qRT-PCR was performed. Because their biological functions were mostly related to the placenta, ten of the fifty DEGs were selected to confirm their expression (Fig. 4). qRT-PCR data confirmed the up-regulation of *FGB*, *FGA*, *FGG*, *SERPINC1*, and *PROC*, as well as the down-regulation of *PLAU, PLAUR, SERPINE1, CD59*, and *CFD* in placenta from IVF-ET samples. Among the 10 tested genes, we observed a significant correlation with the results obtained previously; this confirmed the observed fold changes from our microarray analysis.

### 3.5 Immunohistochemistry

**Figure 5:**
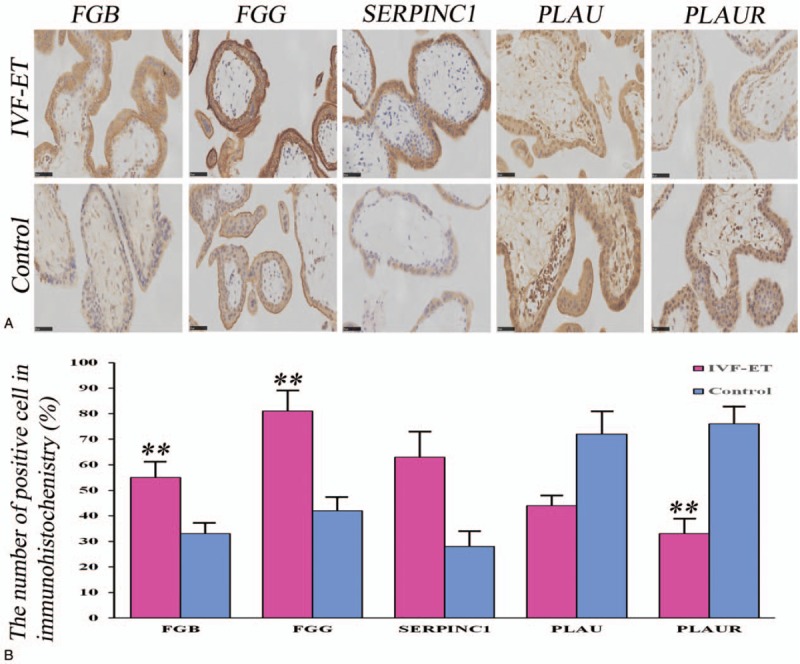
Immunohistochemistry showing the cellular localization of fibrinogen beta chain (FGB), FG gamma chain (FGG), serpin peptidase inhibitor C1 (SERPINC1), plasminogen activator urokinase (PLAU), and PLAU receptor (PLAUR) in placental villus tissues obtained from *in vitro* fertilization-embryo transfer (IVF-ET) and healthy samples in the first trimester. (A) All proteins were located in either the cytoplasm or the cell membrane of trophoblasts. (B) Immunohistochemical analysis of the number of positively stained cells expressing the 5 genes in the IVF-ET and control groups. ∗ indicates *P* < .05; ∗∗ indicates *P* < .01, Scale bar = 50 μm.

To locate differentially expressed proteins related to the complement and coagulation signaling pathways in human placenta during the first trimester, 5 genes, representing different critical functions in these signaling pathways, were selected for IHC analysis: FGB, FGG, SERPINC1, PLAU, and PLAUR. These 5 proteins were found to be located in either the cytoplasm or on the cell membrane of trophoblasts in placental villous tissues (Fig. 5).

## 4 Discussion

There is substantial evidence supporting the hypothesis that several adverse pregnancy outcomes observed after ART are due to suboptimal placentation caused by abnormal trophoblast function.^[23,24]^ Indeed, in humans, after adjusting for several confounding factors, the risk of spontaneous abortion was found to be higher in ART cohorts than in spontaneous pregnancies.^[25]^ Notably, human studies found an increased risk of gestational hypertension, preeclampsia, placenta previa, and placental abruption in ART patients.^[26]^ A number of studies have previously examined and identified alterations in gene expression in placental tissues after IVF-ET treatment.^[27,28]^ A small study investigated the global gene expression in 3 term placentas from IVF-ET pregnancies compared to that in 3 placentas from spontaneous pregnancies. They found 18 DEGs and classified them according to their role in biological process in immune response, transmembrane transport, metabolism, oxidative stress, cell differentiation, and other processes.^[16]^ Furthermore, several studied on the placental transcriptome after IVF-ET in animals have revealed comparable results.^[29]^ The aim of our study was to investigate changes in the complement and coagulation pathways due to ART, and how this would affect placental formation and function to result in placenta-related adverse pregnancy outcomes.

The complement and coagulation cascades are not only parts of the innate immune system, but also effectors of antibody-mediated immunity.^[30]^ The major biological functions of these systems include defense against infections, connecting the innate and adaptive immunity, and the clearance of immune complexes and apoptotic cells.^[31]^ The complement cascade, when activated by the classical, mannose-binding lectin, or alternative pathways, deposits several split products on the cell membrane, ultimately creating a cytotoxic cell lysis complex.^[32]^ The complement split products also include free circulating anaphylatoxins such as C3a and C5a, which can initiate inflammation and tissue injury.^[33]^ Dysregulation or over-activation of these systems are emerging as associated factors in many pregnancy complications.^[34]^ In our study, a total of fifty DEGs related to the complement and coagulation signaling pathways were identified in the placenta during first trimester in pregnancy after IVF-ET therapy compared to placenta samples from natural pregnancies. We mapped these DEGs to the complement and coagulation pathways, and the results showed that these systems were over-activated and uncontrolled. The classical, mannose-binding lectin, and alternative pathways in the complement cascade were all over-activated in early placental tissue after IVF-ET treatment compared to those from natural pregnancy.

In our study, the *C3, C5*, and *CD59* genes, which are known to play a role in the complement cascade, were confirmed to be significantly differently expressed in the placenta between the 2 groups. The expression levels of *C3* and *C5* in IVF-ET placentae were significantly higher compared to placentae from natural pregnancies, while *CD59* expression was significantly lower. The alternative pathway is triggered by the spontaneous hydrolysis of internal thioester bonds within C3 and C5 in the fluid phase, leading to the formation of C3a and C5a. C3a and C5a, which are known as anaphylatoxins, are pleiotropic inflammatory mediators.^[35]^ The complement cascade is controlled by several soluble membrane-bound factors, including CD59, which inhibits the complement pathway at the feto-maternal interface.^[36]^ In a model of spontaneous abortion, C3a and C5a were shown to be required for triggering abortion; C5a in particular was found to be critical for the induction of abortion.^[37]^ Our data were also consistent with those of previous studies in humans investigating C3a, C5a and CD59 levels. A recent study reported that women with unexplained fetal death displayed elevated levels of plasma C3a and C5a compared to those in healthy women.^[38]^ Our research further confirms that this factor at the feto-maternal interface triggered the hyperactivation of the complement cascade after IVF-ET treatment. In addition, similar studies have reported a significant association of elevated C3a and C5a levels and decreased CD59 levels with various pregnancy complications, including gestational hypertension, preterm delivery, and intrauterine growth restrictions.^[39,40]^ Our research adds important evidence that excessive complement activation in complicated pregnancies may be associated with many pre-existing conditions, which are triggered by IVF-ET treatment.

The coagulation system is a component of the homeostatic process and a major contributor to thrombosis. Pregnancy is a physiological hypercoagulable state, and the body must prepare the mother for the hemostatic challenge of delivery.^[41]^ In addition, cytotrophoblast differentiation and fusion to syncytiotrophoblasts requires the initiation of apoptosis and the exposure of negatively charged phospholipids on their membrane surface.^[42]^ The need for the rapid inhibition of hemorrhaging in the placental intervillous spaces during gestation explains the procoagulant nature of trophoblasts.^[43]^ Although these conditions are heterogeneous in their pathophysiology, hereditary and acquired thrombophilia has been shown to be associated with recurrent pregnancy loss and gestational vascular complications.^[44]^ In the present study, KEGG pathway analysis revealed that the coagulation cascade was significantly activated through the intrinsic and extrinsic pathways in placentae after IVF-ET treatment compared to that in placentae from natural pregnancy. In early IVF-ET placentae, a state of hypercoagulability and the local formation of thrombi in the microvasculature draining the site of embryo implantation provide a competent barrier to protect the embryo from the maternal immune system. Apart from its direct role in preventing contact with maternal circulation, the coagulation system can be viewed as an intermediary that converts mechanical information from the embryo implantation site into biochemical signals that trigger cell responses, resulting in vascular biological and inflammatory responses, as well as platelet aggregation.^[45]^ Therefore, an over-activated coagulation system in IVF-ET early placentae may be a protective compensatory mechanism for the survival of the semi-allogeneic fetus. However, IVF-ET treatment may destroy the balance and exceed the compensatory range of the coagulation cascade, resulting in reduced nutrient supply to the embryo and increased thrombophilia-associated pregnancy complications. Moreover, the protein-protein interact network and co-expression analysis derived from STRING database also revealed that some key genes were actively interacted with each other and might be valuable biomarkers and potential novel therapeutic targets against the unfavorable effects of IVF-ET.

To our knowledge, the present study provides evidence for the first time that although the parallel over-activation of the fibrinolysis and complement systems has been observed, the expression of urokinase plasminogen activator (uPA) and urokinase plasminogen activator receptor (uPAR) was significantly reduced at the transcriptional and protein levels in placentae after IVF-ET in the first trimester compared to samples from natural pregnancies. uPA and its receptor uPAR are central molecules for uPA/uPAR/plasmin-dependent proteolysis and plasmin-dependent extracellular proteolysis.^[46]^ To our knowledge, this is the first study to analyze uPA and uPAR expression and localization in the early human placenta after IVF-ET treatment. These distinctive expression patterns were closely associated with their possible individual functions during the IVF-ET implantation process. Decreased expression of uPA and its receptor uPAR is thought to cause impaired trophoblast invasion and expansion, and may also affect the function of massive tissue remodeling in the interstitial endometrium during the process of uterine angiogenesis and degeneration of the epithelial plaque after IVF-ET treatment.^[47]^ Abnormal trophoblast invasion in IVF-ET leads to incomplete uterine vascular conversion, an inadequate fetal blood supply, and a pathological hypoxia milieu. This placental defect after IVF-ET treatment is associated with the persistence of a pro-inflammatory environment and is considered to be a failure of maternal immune tolerance, which is required for normal implantation.

This study examined the molecular mechanisms of IVF-ET-induced alterations to the complement and coagulation signaling pathways in early placenta. We found that the convergence between the complement system and the clotting system extend far beyond the chemical nature of the complement and coagulation pathways, both of which displayed an over-activated proteolytic cascade in early placentae after IVF-ET treatment compared to that in placentae from natural pregnancies. Multiple regulatory loops linking both systems were simultaneously activated to synchronize an effective response by the placenta to disrupt the IVF-ET process. Most often, this cooperative and clearly beneficial effort ensures the elimination of interference by IVF-ET technology to embryo implantation and prevents immediate abortions. However, when some regulatory mechanisms controlling complement activation or hemostasis failure, the complement and coagulation pathways become harmful, significantly contributing to various pregnancy complications, for which only complex therapies targeting multiple molecules can be effective.^[48]^ Indeed, in most case, pregnancies obtained through IVF-ET can be carried to term with no obvious immediate adverse outcomes.^[49]^ This supports the hypothesis that initial defective trophoblast functions could trigger placental adaptive response during pregnancy.

One limitation of this study was the small number of samples investigated in each group. These samples were selected from a cohort of 8 samples collected prospectively for this work. Although the sample size was small, only significantly DEGs (>2-fold) were reported and significant signal pathways were analyzed. These data allow future work to be directed toward the interference of placental formation and function, resulting in placenta-related adverse pregnancy outcomes in IVF-ET. Another limitation was that we could not formally localize the expression of more proteins through IHC, which would improve our understanding of the functional importance of these changes. Further work is needed to determine the biological plausibility of the observed variations in gene expression; however, if our results are consistent with future findings and translate to protein expression in maternal serum, they may still be of value in predicting abnormal outcomes in later pregnancy.

## 5 Conclusion

Our study offers a comprehensive view of dysregulated gene networks in the placental complement and coagulation signaling pathways influenced by IVF-ET treatment, although the detailed regulatory patterns were not explored. Another important result of our study was the discovery of the negative effects on trophoblast invasion, expansion, and tissue remodeling in early placentae after IVF-ET treatment through these 2 systems. Although these interconnections make it difficult to precisely define, separate, and classify biological processes, our data will enable us to focus on a small number of key genes and pathways that need to be better elucidated. Future studies with a larger sample size, focusing on these molecular and biological pathways, may lead to the development of molecular tests to predict adverse outcomes in first trimester. An improved understanding of the equilibrium between complement-mediated immune responses and thrombotic mechanisms triggered by IVF-ET may improve the safety and effectiveness of IVF-ET protocols.

## Author contributions

**Conceptualization:** Liang Zhao.

**Data curation:** Liang Zhao, Lifang Sun, Xiuli Zheng, Rong Zheng, Rui Yang.

**Formal analysis:** Liang Zhao, Lifang Sun, Xiuli Zheng, Jingfang Liu, Rong Zheng, Ying Wang.

**Funding acquisition:** Liang Zhao.

**Investigation:** Liang Zhao, Lifang Sun, Rong Zheng, Rui Yang, Ying Wang.

**Methodology:** Liang Zhao, Lifang Sun, Xiuli Zheng, Jingfang Liu, Rui Yang.

**Project administration:** Liang Zhao, Rong Zheng, Ying Wang.

**Resources:** Liang Zhao.

**Software:** Liang Zhao, Lifang Sun, Xiuli Zheng, Jingfang Liu, Ying Wang.

**Supervision:** Liang Zhao, Lifang Sun, Jingfang Liu, Rui Yang, Ying Wang.

**Validation:** Liang Zhao, Lifang Sun, Xiuli Zheng, Rong Zheng, Ying Wang.

**Visualization:** Liang Zhao, Lifang Sun, Jingfang Liu, Rui Yang.

**Writing – original draft:** Liang Zhao, Lifang Sun, Jingfang Liu, Rong Zheng, Rui Yang.

**Writing – review & editing:** Liang Zhao.

Liang Zhao orcid: 0000-0002-0670-5242.

## Supplementary Material

Supplemental Digital Content
