# Supplementary material for: Alterations in complement and coagulation pathways of human placentae subjected to in vitro fertilization and embryo transfer in the first trimester
Source: Medicine (Baltimore). 2019 Nov 1;98(44):e17031. doi: 10.1097/MD.0000000000017031 (PMC6946305; doi:10.1097/MD.0000000000017031)
Supplement: Supplemental Digital Content [file medi-98-e17031-s001.doc]

Table S1 Primers used for polymerase chain reaction (PCR) experiments.

| Gene Symbol |  | Primer sequence (5`-3`) | Amplicon Size |
| --- | --- | --- | --- |
| FGA | F | 5`-TTTAGAAATATGGATATACCCTC-3` | 500bp |
|  | R | 5`-TAAGAGTGTGTCAGGACATAGAG-3` |  |
| FGB | F | 5`-CTGAAGTCATTCCTAGCAGAG-3` | 316bp |
|  | R | 5`-CAATAAGTCAGAGGTTAACAATT-3` |  |
| FGG | F | 5`-GTGCAAAATCTGGGAACC-3` | 534bp |
|  | R | 5`-AAAGTTACAAGTGCCAGATGC-3` |  |
| Serpinc1 | F | 5`-GAACTGGTCATCAGCCTTTG-3` | 274bp |
|  | R | 5`-CCTGTGAGTCCTTTGGAGGT-3` |  |
| Proc | F | 5`-AAGGATCCATGCTTTTAGGCAT-3` | 327bp |
|  | R | 5`-TATAAGCTTTCATTCCGGTGTAAT-3` |  |
| Plau | F | 5`-TTGGGGGGAGAATTCACTGAG-3` | 367bp |
|  | R | 5`-GGCAGGCAGATGGTCTGTAT-3` |  |
| Plaur | F | 5`-GTGAGGAAGCCCAAGCTACT-3` | 392BP |
|  | R | 5`-ATGTCCAAGGTGGCTTCTTC-3` |  |
| Serpine1 | F | 5`-GTGCTGGTGAATGCCCTCT-3` | 352BP |
|  | R | 5`-GCAGTTCCAGGATGTCGT-3` |  |
| Cd59 | F | 5`-CCGCTCGAGCCGCAGGTTCTGTGGAC-3` | 344bp |
|  | R | 5`-CCGGAATTCGCTTCCTCCGCCTCCTGGT-3` |  |
| Cfd | F | 5`-CGCCTGTACGACGTGCTCCG-3` | 334bp |
|  | R | 5`-GCGGTCCACGACTGGCAAGA-3` |  |
| GAPDH | F | 5`-AGCCCAGCAAGGATACTGAG-3` | 252bp |
|  | R | 5`-GAGGGTGCAGCGAACTTTA-3` |  |

Table S2. Differentially expressed genes in the complement and coagulation pathway are involved in other signaling pathways

| KEGG pathway | ID | gene number | P-Value |
| --- | --- | --- | --- |
| Complement and coagulation cascades | hsa04610 | 50 | 5.49×10^-120^ |
| Staphylococcus aureus infection | hsa05150 | 13 | 2.11×10^-23^ |
| Systemic lupus erythematosus | hsa05322 | 10 | 2.02×10^-13^ |
| Prion diseases | hsa05020 | 7 | 2.03×10^-12^ |
| Pertussis | hsa05133 | 7 | 2.69×10^-10^ |
| Chagas disease (American trypanosomiasis) | hsa05142 | 4 | 6.83×10^-5^ |
| Platelet activation | hsa04611 | 4 | 1.26×10^-4^ |
| Amoebiasis | hsa05146 | 3 | 1.13×10^-3^ |
| Neuroactive ligand-receptor interaction | hsa04080 | 4 | 2.37×10^-3^ |
| Phagosome | hsa04145 | 3 | 3.64×10^-3^ |
| Hematopoietic cell lineage | hsa04640 | 2 | 0.01 |
| AGE-RAGE signaling pathway in diabetic complications | hsa04933 | 2 | 0.01 |
| Transcriptional misregulation in cancer | hsa05202 | 2 | 0.05 |
| Herpes simplex infection | hsa05168 | 2 | 0.05 |
